# Multiple Biventricular Thrombi Associated With Methamphetamine-Associated Cardiomyopathy

**DOI:** 10.31486/toj.21.0097

**Published:** 2022

**Authors:** Syeda H. Zaidi, Umair A. Khan, Shazib Sagheer, Abubaker Sheikh, Mark E. Garcia

**Affiliations:** ^1^ Department of Internal Medicine, Karachi Medical and Dental College, Karachi, Sindh, Pakistan; ^2^ Department of Internal Medicine, University of New Mexico Health Sciences Center, Albuquerque, NM; ^3^ Department of Cardiology, University of New Mexico Health Sciences Center, Albuquerque, NM

**Keywords:** *Cardiomyopathies*, *echocardiography*, *heart failure*, *methamphetamine*, *pulmonary embolism*, *thrombosis*

## Abstract

**Background:** As methamphetamine use has increased around the world, cardiovascular mortality has also increased. Methamphetamine-associated cardiomyopathy (MACM) is one of the serious cardiovascular complications of methamphetamine use. Limited evidence has been published regarding the increased risk of thrombogenicity in the setting of methamphetamine use. We propose that increased thrombogenicity presents a risk factor for intracardiac thrombi.

**Case Report:** A 48-year-old female with a history of MACM was admitted to the hospital with acute decompensated heart failure. Transthoracic echocardiogram revealed multiple biventricular masses requiring further workup, but the patient left against medical advice on warfarin. The patient presented again 2.5 months later with decompensated heart failure. During the second admission, cardiac magnetic resonance imaging (CMR) characterized the masses in the left ventricle as thrombi, and computed tomography of the chest with contrast showed pulmonary embolism. Although the right ventricle mass was not seen on CMR, we believe the mass was a thrombus that either had migrated into the lungs or had resolved with warfarin use.

**Conclusion:** MACM and biventricular thrombi are associated, but the association is rare and not well studied. Although the exact mechanism of this association is unknown, the increased circulating catecholamines are believed to be a contributing factor for increased thrombogenicity in the setting of active methamphetamine use. We suggest keeping a low threshold for surveillance echocardiography to screen for intracardiac thrombi in MACM patients with active methamphetamine use when they present with even mild symptoms of decompensated heart failure.

## INTRODUCTION

Methamphetamine is an addictive psychostimulant drug that acts by promoting the release of catecholamine neurotransmitters in the central nervous system.^1^ Its chronic use is associated with the development of cardiovascular complications such as acute myocardial infarction, aortic dissection, and methamphetamine-associated cardiomyopathy (MACM).^2^ The proposed mechanisms for cardiovascular complications include vasospastic changes induced by methamphetamine, increased catecholamine effects, and direct cardiac toxicity.^2^ Biventricular thrombi in MACM have been rarely reported.^3^ We present the case of a patient with active methamphetamine use and MACM who had a high thrombus burden in both ventricles along with pulmonary embolism.

## CASE REPORT

**Figure 1.**
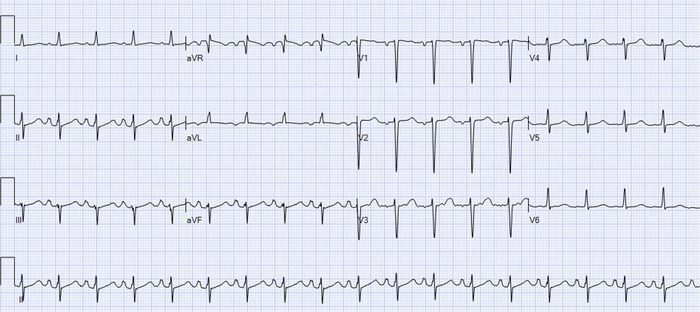
Electrocardiogram shows sinus tachycardia with a heart rate of 109/min, left atrial enlargement, and left axis deviation.

A 48-year-old female with a history of known MACM and chronic systolic heart failure with a left ventricular ejection fraction of 20% was admitted to the hospital with acute decompensated systolic heart failure. Other relevant history included active tobacco use disorder, active methamphetamine use, hypothyroidism, and frequent heart failure hospitalizations because of dietary and medication noncompliance. Family history was negative for thrombophilia. Initial vital signs were significant for tachycardia with a heart rate of 120/min, borderline hypotension with blood pressure of 89/90 mm Hg, and tachypnea with a respiratory rate of 28/min. Laboratory results were significant for troponin elevation of 0.208 ng/mL (reference, <0.060 ng/mL). Workup for nonischemic cardiomyopathy was unrevealing. Ferritin, iron, iron saturation, thyroid-stimulating hormone, antinuclear antibody, hepatitis C antibody, urine and serum protein electrophoresis, and HIV screen were normal. Because antinuclear antibody was normal, further workup for lupus was not pursued. Hypercoagulable workup with rotational thromboelastometry was also normal. Electrocardiogram was without ischemic changes but showed QTc prolonged at 520 milliseconds and left atrial enlargement (Figure 1).

Two years prior, coronary angiography had shown normal coronary arteries.

**Figure 2.**
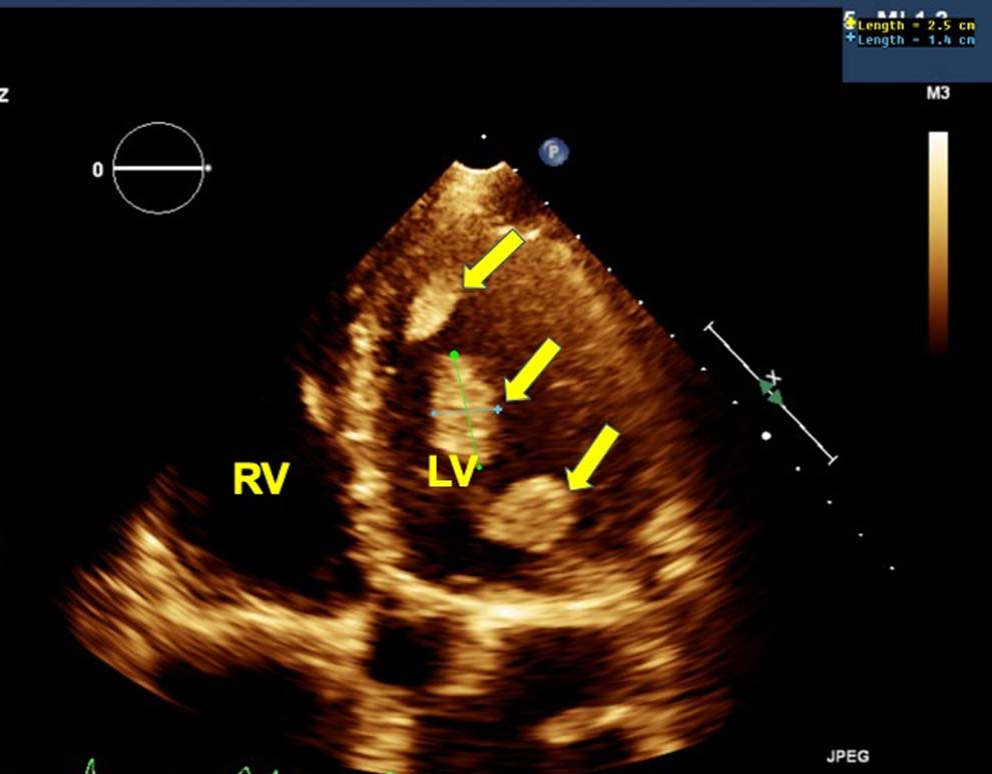
**Transthoracic echocardiogram apical 4-chamber view demonstrates multiple left ventricle masses (arrows) with the largest mass measuring 2.5 cm × 1.5 cm.** LV, left ventricle; RV, right ventricle.

**Figure 3.**
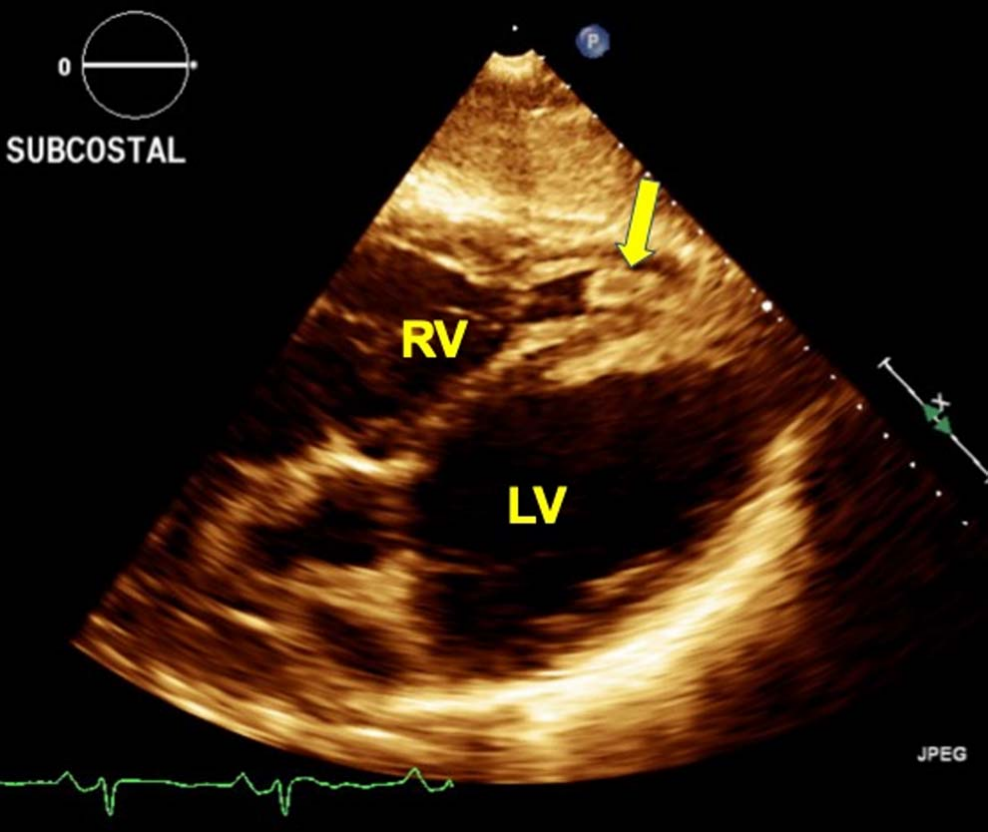
**Transthoracic echocardiogram subcostal view demonstrates right ventricle mass close to the apex (arrow).** LV, left ventricle; RV, right ventricle.

Transthoracic echocardiogram (TTE) showed left ventricular ejection fraction of 18%, severely dilated left ventricle with left ventricular internal dimension at end diastole index of 3.93 cm/m^2^, right ventricle of normal size, grade III diastolic dysfunctions, severe global hypokinesis, and at least 4 masses in the left ventricle and 1 mass in the right ventricle (Figures 2 and 3). Because of suspicion that the multiple masses were thrombi, the patient was started on anticoagulation with high-intensity intravenous heparin continuous infusion. The heparin dose was titrated to achieve adequate therapeutic anticoagulation as guided by anti-factor Xa level (therapeutic range, 0.30-0.70 IU/mL). The biventricular masses had some features not convincing for thrombi, such as multiple masses, presence in both ventricles, and cystic consistency. Cardiac magnetic resonance imaging (CMR) was planned for further characterization, but the patient left against medical advice after 3 days of hospitalization. At discharge, the patient was given oral warfarin 2.5 mg to be taken once daily for a minimum of 3 months.

**Figure 4.**
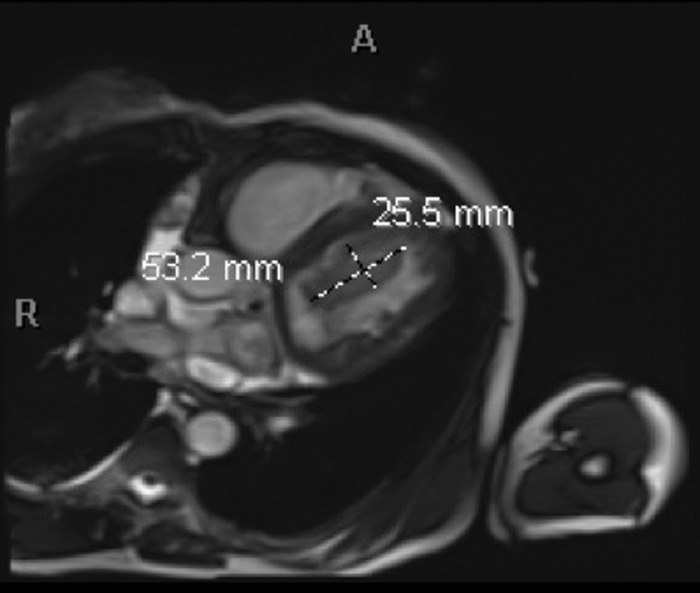
Cardiac magnetic resonance imaging with true fast imaging with steady-state free precession (TRUFI) sequence showing oblong left ventricle mass along the basal and mid anterior wall measuring approximately 5.3 mm × 2.5 mm in the 4-chamber view. The signal of this mass is slightly higher than the myocardium on the TRUFI precontrast images and is a high signal on the T2-weighted images.

Approximately 2.5 months later, the patient presented again with decompensated heart failure. At this time, she presented with a heart rate of 130/min and hypoxia requiring 4 L oxygen. Computed tomography (CT) chest with contrast revealed right lower lobe segmental pulmonary embolism. She was initially started on therapeutic dose heparin and was later switched to warfarin. Repeat TTE at this admission showed severely reduced left ventricular systolic function with ejection fraction of 18% and 2 left ventricular masses. CMR obtained during this admission characterized the left ventricular masses as thrombi but showed no evidence of malignancy or infection (Figure 4). Right ventricular thrombus was not seen on CMR in the setting of intermittent warfarin use for approximately 2.5 months. One clinical hypothesis was that the patient's warfarin use could have contributed to a decrease in her left ventricular thrombi burden and resolution of the right ventricular thrombus. Another possibility was that the right ventricular mass noted on TTE during the first admission, which the clinicians believed was a thrombus based on its appearance on the echocardiogram (Figure 3), could have migrated to the lungs, causing pulmonary embolism. After optimization of the patient's heart failure, she was discharged on warfarin 2.5 mg orally once daily for 3 months with follow-up appointments with the anticoagulation clinic, heart failure clinic, and primary care physician. She continued to refuse referral to a substance use clinic in the setting of active methamphetamine use.

## DISCUSSION

Cardiovascular complications of methamphetamine use are well established and include malignant hypertension, arrhythmias, aortic dissection, myocardial infarction secondary to vasospasm, stroke, pulmonary arterial hypertension, and cardiomyopathy.^2^ The incidence of MACM among methamphetamine users increased from 1.8 percent in 2009 to 5.6 percent in 2014.^4^ The underlying mechanisms include the replacement of myocytes by fibrosis and myocardial contractile dysfunction caused by a combination of catecholamine-induced myocardial wall stress and methamphetamine-induced direct myocardial cell toxicity.^2,5^

Patients with MACM tend to be younger than patients with cardiomyopathy attributable to other causes. Patients typically present with severe heart failure, reduced ejection fraction, and compensatory tachycardia in the setting of low cardiac output. The usual echocardiogram findings are severe left ventricular dilatation, reduced systolic function, right ventricle dilatation, and global left ventricle hypokinesis.^2,6^ Patients with MACM have a 33% prevalence of developing left ventricular thrombus and a 3.3% prevalence for developing right ventricular thrombus,^5^ likely attributable to a combination of increased catecholamine activation, prothrombotic state, and severe cardiac dysfunction.^2^ Sympathetic catecholamines have been shown to cause platelet aggregation in vitro and to enhance thrombus formation in plastic shunts in vivo.^7^ The aforementioned factors contribute to the increased risk of intracardiac thrombus burden seen in MACM patients.

In our case, we believe the right ventricular mass seen on TTE during the initial hospitalization was a thrombus as its appearance and echocardiogram density were similar to the echocardiogram density of the masses seen in the left ventricle. We believe the reason the right ventricular mass was not seen on CMR during the second admission is because the thrombus was small and had either resolved with anticoagulation or migrated into the lungs, causing the pulmonary embolism that was seen on the CT chest. To the best of our knowledge, ours is the second reported case of biventricular thrombi in the setting of MACM.^3^ However, compared to the case reported by Janardhanan and Kannan,^3^ our case is unique in several ways. First, biventricular thrombi associated with MACM are rarely reported, especially with simultaneous pulmonary embolism. We believe the right ventricular thrombus migration was the cause of pulmonary embolism in our patient, which highlights the instability of the thrombus. Second, given the patient's tachycardia and borderline hypotension, we repeated the TTE during the second hospitalization as opposed to the usual management of not obtaining TTE during heart failure–related readmissions. This management approach was a judgment call that perhaps prevented a fatal event, such as pulseless electrical activity cardiac arrest from pulmonary embolism or embolic phenomenon including stroke, by starting the patient on anticoagulation during her first hospitalization. Based on the limited evidence and our case, we suggest prophylactic anticoagulation for patients who are actively using methamphetamine and have MACM, severely depressed ejection fraction, and severe left ventricle dilation.

## CONCLUSION

Based on the literature and methamphetamine pathophysiology, we propose that in addition to the severe dilated cardiomyopathy, active methamphetamine use is not only a risk factor for intracardiac thrombi but can also lead to increased thrombus burden with biventricular involvement. Perhaps elusive mechanisms are involved that need to be investigated further. We suggest repeating TTE in patients with MACM and active methamphetamine use during every hospitalization as a surveillance strategy for biventricular thrombi, especially if a left heart catheterization is being considered, as many of these patients undergo this procedure. Anticoagulation with warfarin is advised as the mainstay of therapy to treat and potentially prevent the recurrence of the thrombi.

## Notes

*This article meets the Accreditation Council for Graduate Medical Education and the American Board of Medical Specialties Maintenance of Certification competencies for Patient Care and Medical Knowledge.*
